# ING5 suppresses proliferation, apoptosis, migration and invasion, and induces autophagy and differentiation of gastric cancer cells: a good marker for carcinogenesis and subsequent progression

**DOI:** 10.18632/oncotarget.3735

**Published:** 2015-03-30

**Authors:** Wen-feng Gou, Dao-fu Shen, Xue-feng Yang, Shuang Zhao, Yun-peng Liu, Hong-zhi Sun, Rong-jian Su, Jun-sheng Luo, Hua-chuan Zheng

**Affiliations:** ^1^ Cancer Research Center, Key Laboratory of Brain and Spinal Cord Injury of Liaoning Province, and Laboratory Animal Center, The First Affiliated Hospital of Liaoning Medical University, Jinzhou, China; ^2^ Department of Oncological Medicine, The First Affiliated Hospital of China Medical University, Shenyang, China; ^3^ Experimental Center, Liaoning Medical University, Jinzhou, China

**Keywords:** gastric cancer, ING5, aggressive phenotypes, progression, carcinogenesis

## Abstract

Here, we found that ING5 overexpression increased autophagy, differentiation, and decreased proliferation, apoptosis, migration, invasion and lamellipodia formation in gastric cancer cells, while ING5 knockdown had the opposite effects. In SGC-7901 transfectants, ING5 overexpression caused G_1_ arrest, which was positively associated with 14-3-3 overexpression, Cdk4 and c-jun hypoexpression. The induction of Bax hypoexpression, Bcl-2, survivin, 14-3-3, PI3K, p-Akt and p70S6K overexpression by ING5 decreased apoptosis in SGC-7901 cells. The hypoexpression of MMP-9, MAP1B and flotillin 2 contributed to the inhibitory effects of ING5 on migration and invasion of SGC-7901 cells. ING5 overexpression might activate both β-catenin and NF-κB pathways in SGC-7901 cells, and promote the expression of down-stream genes (*c-myc*, *VEGF*, *Cyclin D1*, *survivin*, and *interleukins*). Compared with the control, ING5 transfectants displayed drug resistance to triciribine, paclitaxel, cisplatin, SAHA, MG132 and parthenolide, which was positively related to their apoptotic induction and the overexpression of chemoresistance-related genes (*MDR1*, *GRP78*, *GRP94*, *IRE*, *CD147*, *FBXW7*, *TOP1*, *TOP2*, *MLH1*, *MRP1*, *BRCP1* and *GST-π*). ING5 expression was higher in gastric cancer than matched mucosa. It was inversely associated with tumor size, dedifferentiation, lymph node metastasis and clinicopathological staging of cancer. ING5 overexpression suppressed growth, blood supply and lung metastasis of SGC-7901 cells by inhibiting proliferation, enhancing autophagy and apoptosis in xenograft models. It was suggested that ING5 expression might be employed as a good marker for gastric carcinogenesis and subsequent progression by inhibiting proliferation, growth, migration, invasion and metastasis. ING5 might induce apoptotic and chemotherapeutic resistances of gastric cancer cells by activating β-catenin, NF-κB and Akt pathways.

## INTRODUCTION

Loss (Class I) and inactivation (Class II) of tumor suppressor genes (TSG) result from chromosomal deletion, mutation or hypermethylation and in immortality of cancer cells [1]. The inhibitor of growth (ING) 5 functions as Class II TSG due to a suppressive role in initiation, promotion and development of tumors [2]. ING5 consists of several different domains, among which LZL (leucine zipper like) has been shown to promote DNA repair, apoptosis and chromatin remodeling, NLS (nuclear localization signal) to guide nuclear translocation, and NCR (novel conserved region) to remodel chromatin. ING5 can interact with histone acetyl transferase (HAT) complexes (H4-HBO1-JADE-ING5 and H3-MOZ-MORF-BRPF-ING5) [3, 4]. H4-HBO1–JADE–ING5 HAT complex might enhance DNA replication in cooperation with the mini-chromosome maintenance complex because ING5 knockdown abolishes DNA synthesis and ING5 overexpression decreases S-phase cells [5, 6]. ING5 transcriptionally induces the expression of p21/waf1 and interacts with Cyclin A1 inhibitor to suppress cell cycle progression [7, 8]. Liu et al. [8] reported that ING5 assisted Tip60 in acetylating p53 at K120 in response to DNA damage by a complex formation with p53 and Tip60. The acetylated ING5 subsequently bound to the promoters of its target apoptotic genes, *Bax* and *GADD45*.

ING5 deletion, mutation and down-regulation were detectable in oral carcinogenesis [9, 10]. The reduction in nuclear ING5 expression and its cytoplasmic translocation were observed in head and neck squamous cell cancer (HNSCC) [11], and positively linked to tumorigenesis and aggressive behaviors of colorectal or gastric cancers [12, 13]. In the present study, we observed the *in vivo* and *vitro* effects of altered ING5 expression on proliferation, apoptosis, autophagy, differentiation, invasion, migration, metastasis and chemoresistance of gastric cancer cells, and analyzed the relevant molecular mechanisms. ING5 expression was also compared with carcinogenesis and aggressive behaviors of gastric cancer.

## RESULTS

### *ING5* expression in gastric cancer cells

**Figure 1:**
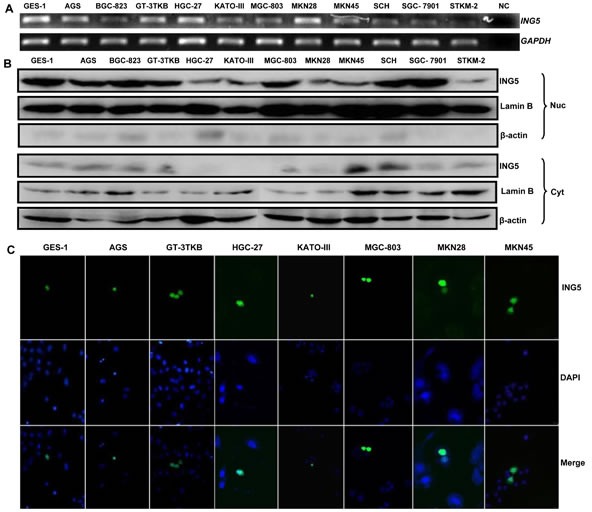
ING5 expression and localization in gastric cancer and epithelial cell lines **A**: Different amplicons of *ING5* mRNA were detected and showed inconsistent density in all gastric cancer and epithelial cells with an internal control of *GAPDH*. **B**: Cytosolic (Cyt) and nuclear (Nuc) fraction proteins were loaded and probed with anti-human ING5 antibody (28kDa) with β-actin (42 kDa) or Lamin B (60kDa) as an internal control. **C**: GES-1, AGS, GT-3TKB, HGC-27, KATO-III, MGC-803, MKN28 and MKN45 cells were transiently transfected with pEGFP-N1-ING5 plasmid and observed under confocal microscope (green for ING5; DAPI, blue for nucleus). NC, negative control.

**Figure 2:**
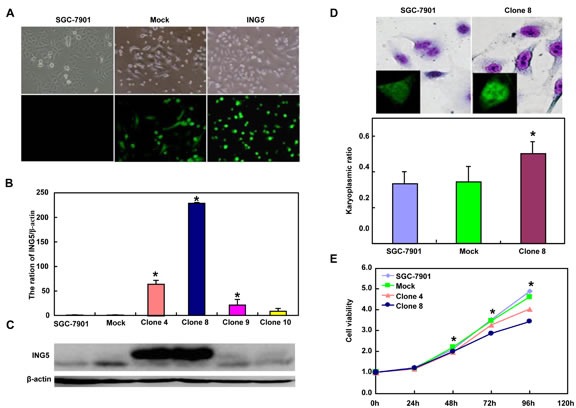

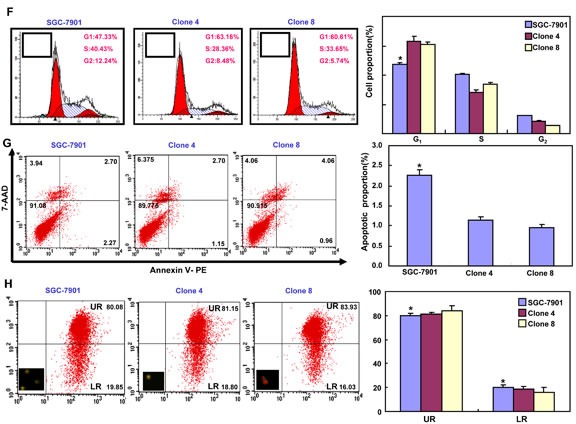

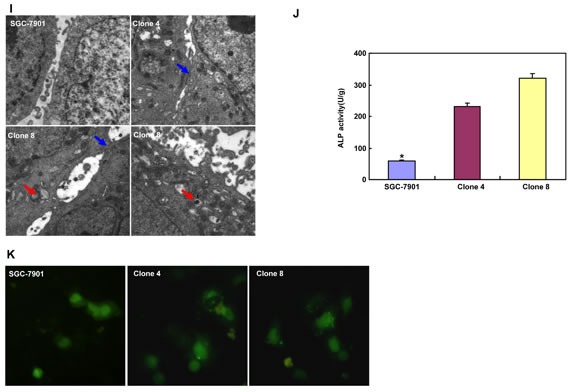

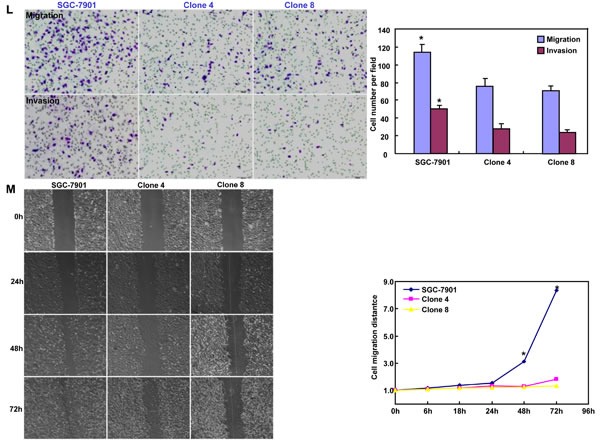

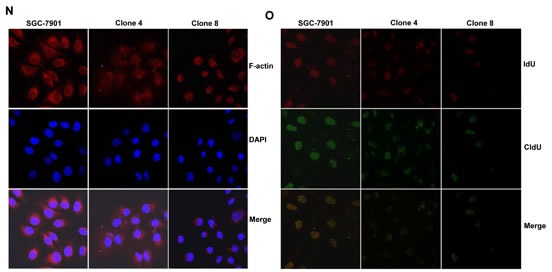

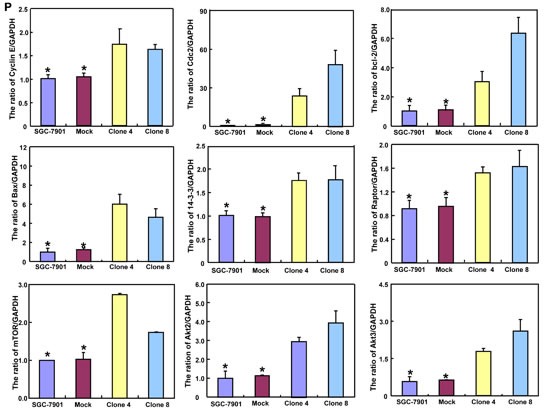

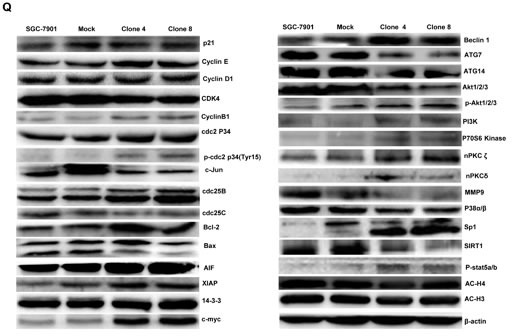

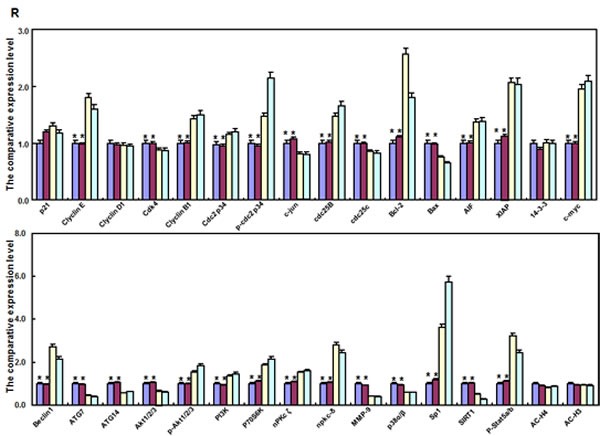
ING5 overexpression altered the phenotypes and the expression of their relevant genes of SGC-7901 cells After transfection of pEGFP-N1-ING5, ING5 expression became strong in SGC-7901 cells by morphological examination (green: GFP-fused ING5), RT-PCR and Western blot (**A**-**C**). The transfectants showed a high karyoplasmic ratio (**D**), low growth (E), and G_1_ arrest (**F**) in comparison to the control. An apoptosis-resistance and a low mitochondrial potential was detected in ING5 transfectants in comparison to the maternal cells, evidenced by Annexin V assay (**G**) and JC-1 staining (**H**). There was a better differentiation in ING5 transfectants than the control according to the tight junction of TEM (blue arrows, **I**) and ALP activity (**J**). The higher autophagy was detectable in SGC-7901 transfectants than the maternal cells, evidenced by autophasome of transmission electron microscope (red arrows) and punctate LC3B-EGFP (**K**). Compared with the control, ING5-overexpressing SGC-7901 had a weak ability to migrate and invade by transwell chamber assay (**L**) and wound healing (**M**), and showed a weak lamellipodia formation, labeled with F-actin immunostaining (**N**). There were a lower proportion of S-phase cells in SGC-7901 transfectants than the control by IdU and CIdU staining (**O**). The phenotype-related genes were screened by real-time PCR (**P**) and Western blot (**Q** and **R**). *p < 0.05, compared with the transfectants; UR, upper right; LR, low right.

*ING5* mRNA was strongly expressed in GES-1, AGS, GT-3 TKB, HGC-27, MKN28, but weakly in BGC-823, KATO-III, MGC-803, MKN45, SCH, SGC-7901, and STKM-2 (Figure 1A). Western blot showed ING5 overexpression in the nuclei of GES-1, AGS, BGC-823, GT-3 TKB, MGC-803, SCH and SGC-7901, but weak in HGC-27, KATO-III, MKN-28, MKN-45, and STKM-2 (Figure 1B). Transient transfection indicated that GFP-fused ING5 was localized in the nuclei of GES-1, AGS, GT-3TKB, HGC-27, KATO-III, MGC-803, MKN28, MKN45 and SGC-7901 (Figure 1C and Figure 2A).

### The effects of altered ING5 expression on biological phenotypes or their relevant molecules of gastric cancer cells

After transfected with pEGFP-N1-ING5, SGC-7901 cells overexpressed *ING5* at both mRNA and protein levels (Figure 2A-2C). The high karyoplasmic ratio, slow growth and G_1_ arrest were seen in ING5 transfectants, compared with the control (Figure 2D-2F, *p* < 0.05). There was a lower apoptosis evidenced by Annexin-V (Figure 2G, *p* < 0.05), a higher mitochondrial membrane potential by JC-1 staining (Figure 2H, *p <* 0.05) and a better differentiation by tight junction (Figure 2I) and alkaline phosphatase (ALP) activity (Figure 2J, *p* < 0.05) in SGC-7901 transfectants than the control. SGC-7901 transfectants showed more autophasomes (Figure 2I) and stronger punctate LC3B-EGFP (Figure 2K) than the control. ING5 overexpression could suppress migration and invasion by transwell chamber assay (Figure 2L, *p* < 0.05) or wound healing (Figure 2M, *p* < 0.05), and weaken lamellipodia formation, labeled with F-actin staining (Figure 2N) in comparison to the control or mock. ING5 transfectants showed lower DNA replication than SGC-7901 by IdU and CIdU staining (Figure 2O).

As shown in Figure 2P, ING5 transfectants displayed the overexpression of *Cyclin E, cdc2, Bcl-2, Bax, 14-3-3, Raptor, mTOR, Akt2* and *Akt3,* compared with the control and mock by real-time PCR (*p* < 0.05). At the protein level (Figure 2Q and 2R), ING5 overexpression increased the expression of Cyclin E, Cyclin B1, cdc-2, p-cdc2 p34, cdc25B, Bcl-2, AIF, XIAP, c-myc, Beclin 1, p-Akt1/2/3, PI3K, p70S6K, nPKC ζ, nPKCδ, Sp1, and p-stat5a/b (*p* < 0.05), but decreased the expression of Cdk4, c-jun, cdc25c, Bax, ATG7, ATG14, Akt1/2/3, MMP-9, p38 and SIRT1 in SGC-7901 cells (*p* < 0.05).

**Figure 3:**
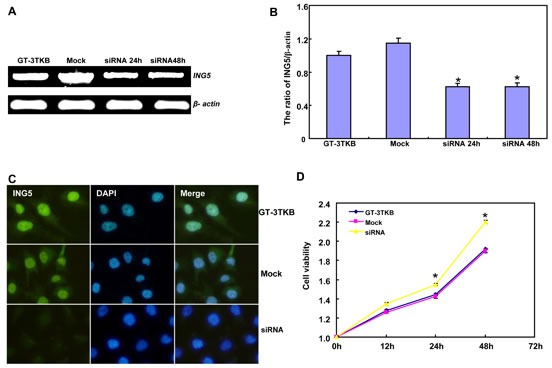

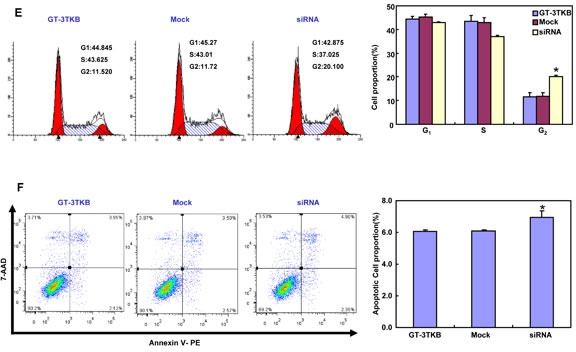

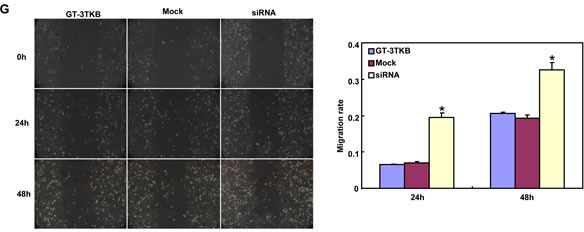

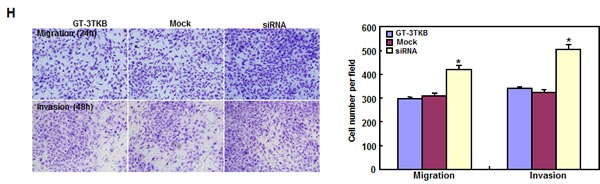

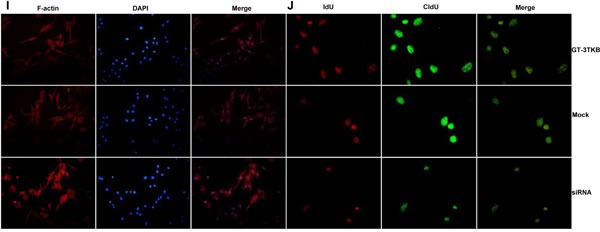
The effects of ING5 knockdown on the aggressive phenotypes of GT-3TKB cells After siRNA transfection, ING5 expression was reduced in GT-3 TKB cells by RT-PCR (**A**), densitometric quantification (**B**) and immunofluorescence (**C**). The transfectants showed a faster growth (**D**), less S and more G_2_ cells (**E**) than the control and mock. There was an apoptosis-induced effect of ING5 hypoexpression in GT-3TKB transfectant, evidenced by Annexin V assay (**F**). ING5 siRNA-treated GT-3TKB had a stronger ability to migrate, invade (**G**, **H**) and form lamellipodia (**I**) than the control and mock. The ratio of S-phase cells was reduced in GT-3TKB transfectants by IdU and CIdU staining (**J**) in comparison to the control and mock. *p < 0.05, compared with the control and mock.

siRNA treatment significantly reduced ING5 expression in GT-3TKB by RT-PCR and immunofluorescence respectively (Figure 3A-3C). ING5 knockdown could enhance proliferation, cell cycle progression, apoptosis, migration, invasion and lamellipodia formation (Figure 3D-3I, *p* < 0.05). IdU and CIdU staining showed a low ratio of S-phase cells in GT-3TKB transfectants (Figure 3J).

### ING5 overexpression activated β-catenin and NF-κB pathway in gastric cancer cells

**Figure 4:**
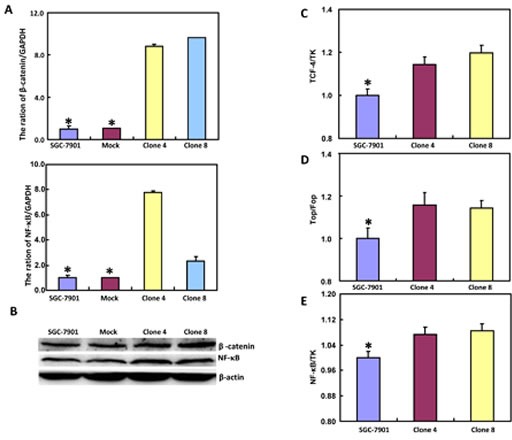

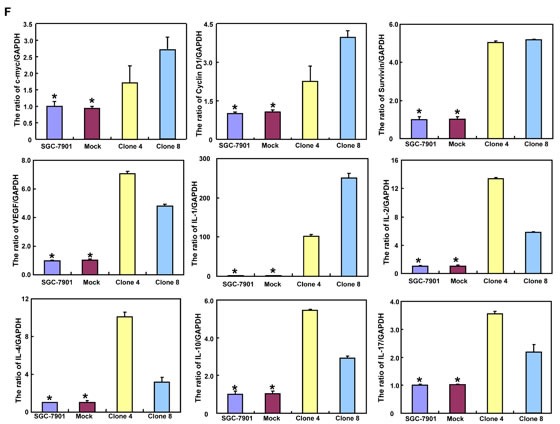
ING5 expression up-regulates both β-catenin and NF-κB signal pathway in SGC-7901 cells After transfection of pEGFP-N1-ING5, SGC-7901 transfectants more expressed the mRNA and protein of β-catenin and NF-κB than mock and control by RT-PCR (**A**) and Western blot (**B**), respectively. There was a high activity of TCF-4 (**C**) and NF-κB (**E**) promoters, TCF-4-mediated transcription (**D**) in ING5 transfectants, compared with the control and mock. The down-stream genes of both β-catenin and NF-κB were detected by real-time PCR (F). *p < 0.05, compared with the transfectants.

The mRNA and protein expression of β-catenin was increased in SGC-7901 transfectants, compared with the control and mock (Figure 4A and 4B, *p <* 0.05). Dual luciferase reporter gene assay demonstrated that both TCF-4 promoter activity and TCF4-mediated gene transcription activity became higher in SGC-7901 transfectants than the control (Figure 4C and 4D, *p* < 0.05). It was the same for the down-stream target genes, such as *c-myc*, *Cyclin D1*, *survivin* and *VEGF* (Figure 4F, *p* < 0.05). Additionally, promoter activity and expression level of NF-κB were higher in SGC-7901 transfectants than the control or mock (Figure 4A, 4B and 4E, *p* < 0.05). It was the same for the mRNA expression of its down-stream target genes, including *IL-1*, *-2*, *-4*, *-10*, and *-17* (Figure 4F, *p* < 0.05).

### ING5-mediated chemotherapeutic resistance of SGC-7901 cells

**Figure 5:**
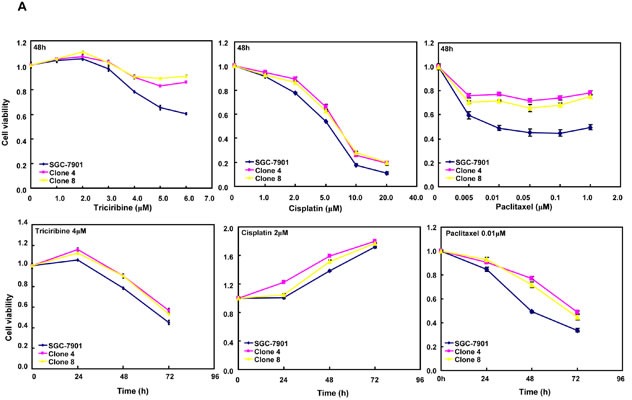

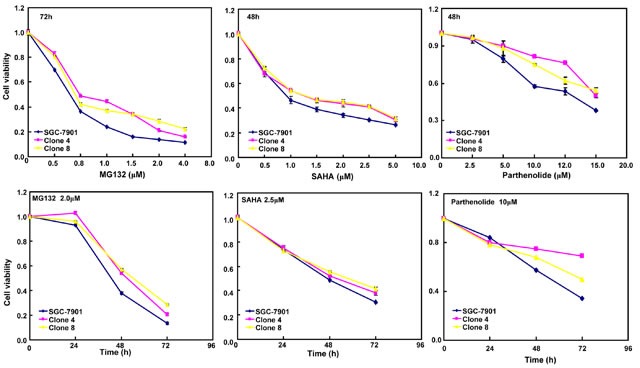

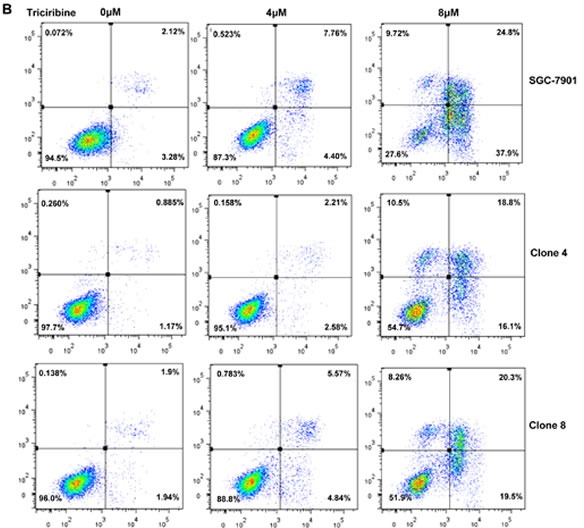

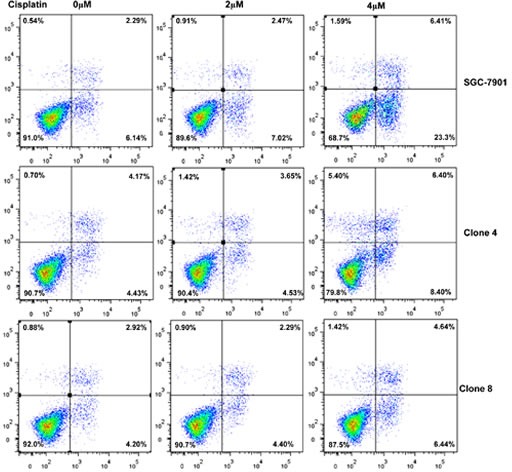

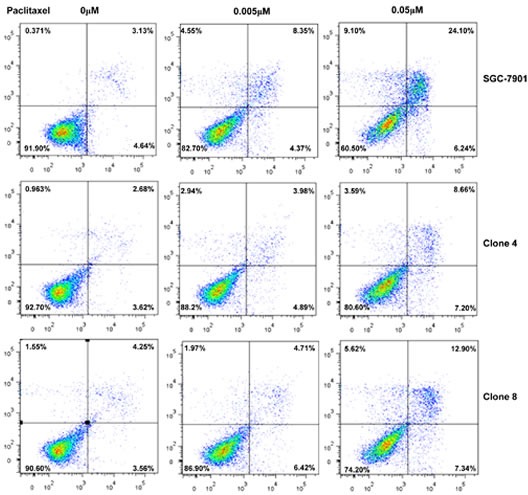

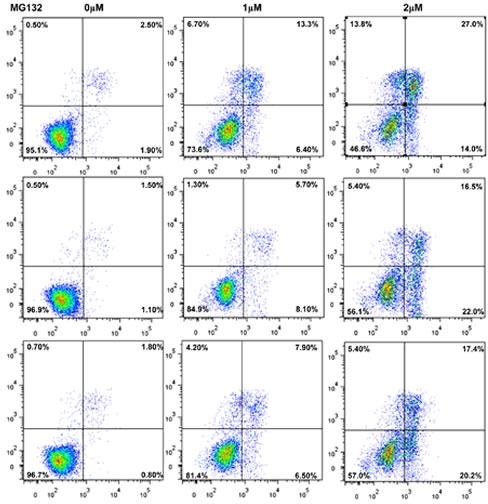

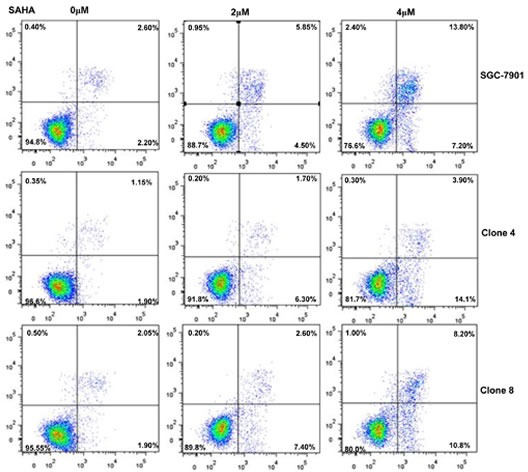

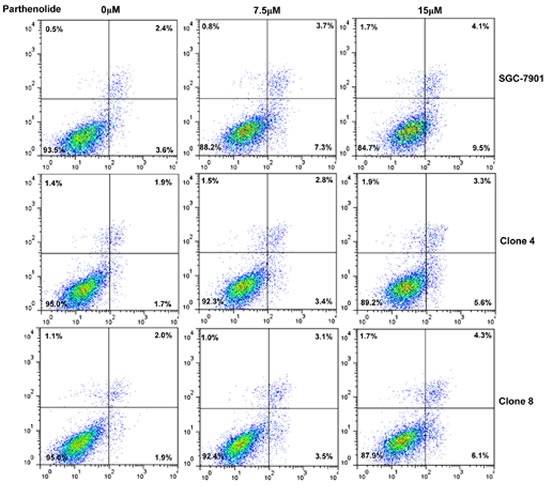

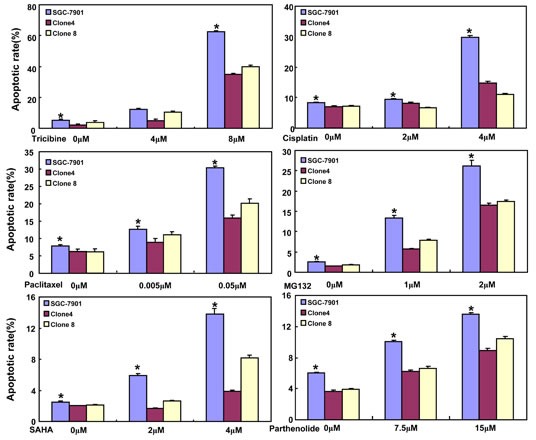

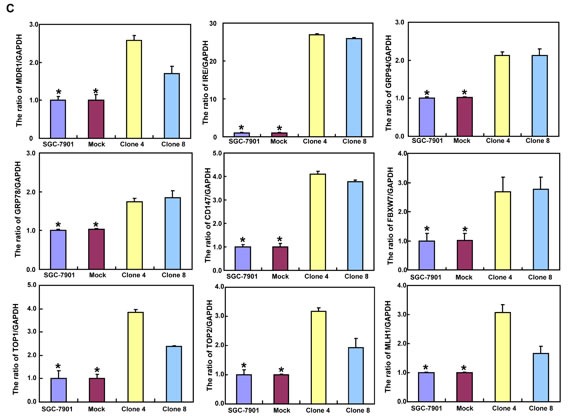

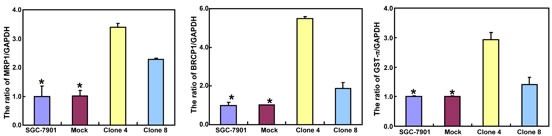
ING5 overexpression attenuated the sensitivity of SGC-7901 to chemotherapeutic agents After exposed to triciribine, paclitaxel, cisplatin, SAHA, MG132 and parthenolide, ING5 transfectants showed a higher viability and a lower apoptotic level than the control in both concentration and time-dependent manners (**A** and **B**). The chemoresistance-related genes were screened by real-time RT-PCR (**C**). *p < 0.05, compared with the transfectants.

After the exposure to triciribine, paclitaxel, cisplatin, SAHA, MG132 and parthenolide, SGC-7901 transfectants showed higher viability and lower apoptosis than the control in both dose- and time-dependent manners (Figure 5A and 5B, *p* < 0.05). The transfectants more expressed *MDR1*, *GRP78*, *GRP94*, *IRE*, *CD147*, *FBXW7*, *TOP1*, *TOP2*, *MLH1*, *MRP1*, *BRCP1*, and *GST-π* than the mock and control by real-time RT-PCR (Figure 5C, *p* < 0.05).

### Functional network, gene ontology and canonical pathway analysis for ING5 overexpression in SGC-7901 cells

**Figure 6:**
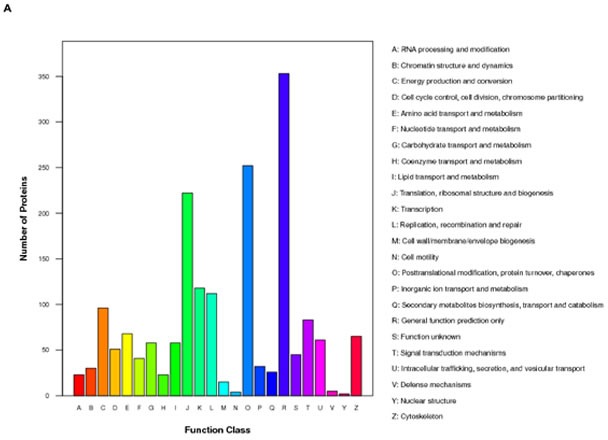

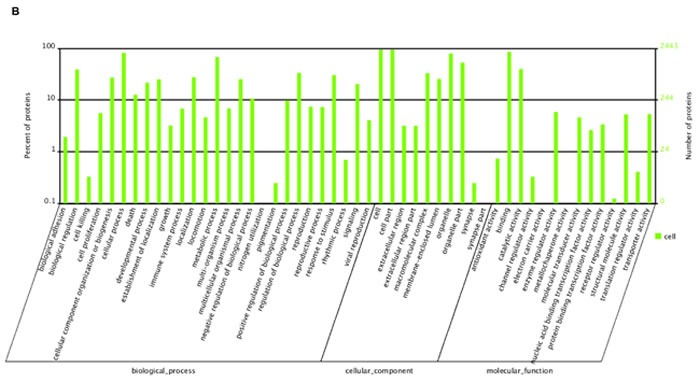

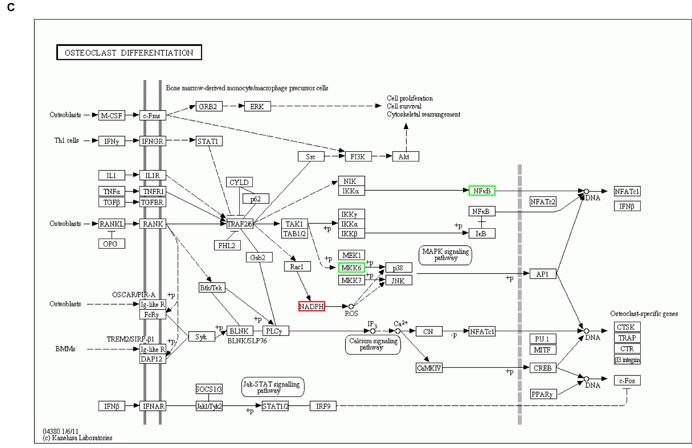

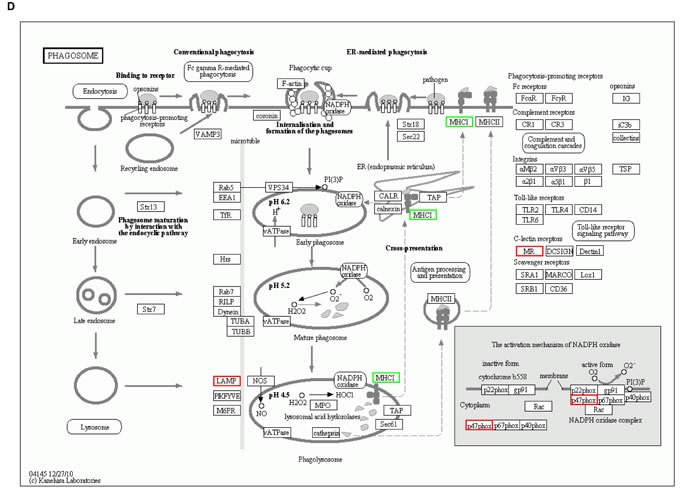
The protein expression profiles in ING5 transfectants of SGC-7901 cells **A**: COG (Cluster of Orthologous Groups of proteins) function classification of cell sequence. **B**: Gene ontology includes molecular function, cellular component, and biological process. **C**: Osteoclast differentiation of KEGG (Kyoto Encyclopedia of Genes and Genomes) Pathway. **D**: Phagosome of KEGG Pathway.

iTRAQ-labeling LC-MS/MS analyses identified ∼197 proteins in SGC-7901 transfectants as shown in Table 1 (up-regulated) and Table 2 (down-regulated). We analyzed the top networks and canonical pathways according to more than 2-fold increase or decrease in expression. As indicated in Figure 6A, COG (cluster of orthologous groups of proteins) functions mainly are composed of general function prediction only (19.2%), posttranslational modification, protein turnover, chaperones (13.7%), and translation, ribosomal structure and biogenesis (12.1%). Biological processes contained cellular process (17.0%), metabolic process (14.0%), and biological regulation (8.1%), cell components did organelle (34.2%) and macromolecular complex (8.5%), and cell function did binding (55.7%) and catalytic activity (25.7%, Figure 6B). The top canonical pathways analyses indicated significant differences in osteoclast differentiation (Figure 6C), phagosome (Figure 6D), cell adhesion molecules, amyotrophic lateral sclerosis, ECM-receptor interaction and so forth (Table 3, *p* < 0.05).

**Table 1:** The up-regulated genes in ING5-overexpressing SGC-7901 cells

Num	Hit Num	Description	Score	Mass	Ratio
1	1016	PLOD3 Procollagen-lysine,2-oxoglutarate 5-dioxygenase 3	145	93211	0.664
2	53	HIST2H2BE Histone H2B type 2-E	2729	20300	0.614
2	53	HIST1H2BO Histone H2B type 1-O	2729	20286	
2	53	HIST1H2BJ Histone H2B type 1-J	2729	20284	
2	53	HIST1H2BB Histone H2B type 1-B	2729	20330	
3	2077	MRPL24 39S ribosomal protein L24, mitochondrial	48	30185	0.501
4	898	NFKB2 Isoform 4 of Nuclear factor NF-kappa-B p100 subunit	167	109166	0.451
4	898	NFKB2 Isoform 1 of Nuclear factor NF-kappa-B p100 subunit	167	109237	
5	695	S100A13 Protein S100-A13	231	15723	0.662
6	402	VTN Vitronectin	446	61458	0.432
6	402	SEBOX cDNA FLJ51266, highly similar to Vitronectin	446	27412	
7	599	HLA-B;HLA-C;HLA-A;MICA;LOC441528 MHC class I antigen (Fragment)	278	22123	0.527
8	2052	SDC1 Syndecan-1	50	35598	0.654
9	2226	MAP2K6 Isoform 1 of Dual specificity mitogen-activated protein kinase kinase 6	42	46937	0.662
9	2226	MAP2K6 Putative uncharacterized protein DKFZp686N0154	42	21680	
10	703	APOA1 Apolipoprotein A-I	227	37756	0.234
10	703	APOA1 Uncharacterized protein	227	34089	

**Table 2:** The down-regulated genes in ING5-overexpressing SGC-7901 cells

Num	Hit Num	Description	Score	Mass	Ratio
1	1259	PIGK GPI-anchor transamidase	105	51592	1.793
1	1259	PIGK Uncharacterized protein	105	41393	
2	376	PPP1R2 Protein phosphatase inhibitor 2	494	28534	1.636
2	376	PPP1R2P3 Putative protein phosphatase inhibitor 2-like protein 3	494	28567	
2	376	PPP1R2 Uncharacterized protein	494	24100	
3	546	SNORD19B;GNL3 Isoform 2 of Guanine nucleotide-binding protein-like 3	311	80181	1.837
3	546	SNORD19B;GNL3 Isoform 1 of Guanine nucleotide-binding protein-like 3	311	83154	
4	439	PACSIN3 Protein kinase C and casein kinase substrate in neurons 3, isoform CRA_b	404	56506	1.571
4	439	PACSIN3 cDNA FLJ61415, highly similar to Protein kinase C and casein kinase substratein neurons protein 3	404	56247	
5	899	UBXN7 UBX domain-containing protein 7	167	63441	1.625
5	899	UBXN7 Uncharacterized protein	167	45554	
6	1003	MAP1B Microtubule-associated protein 1B	147	342545	1.666
7	839	FAM129A Protein Niban	181	121683	2.236
8	920	CAT Catalase	162	69377	1.578
9	1665	TIMM50 Isoform 2 of Mitochondrial import inner membrane translocase subunit TIM50	70	57335	1.508
9	1665	TIMM50 Isoform 1 of Mitochondrial import inner membrane translocase subunit TIM50	70	45021	
10	2208	CCAR1 Isoform 1 of Cell division cycle and apoptosis regulator protein 1	43	163844	1.622
10	2208	CCAR1 Isoform 2 of Cell division cycle and apoptosis regulator protein 1	43	162067	
10	2208	CCAR1 120 kDa protein	43	147501	
11	1475	ERO1L ERO1-like protein alpha	84	64340	1.601
12	1103	LAMP1 Lysosome-associated membrane glycoprotein 1	126	51147	1.507
13	604	VBP1 von Hippel-Lindau binding protein 1, isoform CRA_b	276	34295	1.537
13	604	VBP1 Uncharacterized protein	276	29079	
14	1190	SH3PXD2B SH3 and PX domain-containing protein 2B	115	124788	1.541
15	1291	FLOT2 Flotillin-2	101	58385	1.617
15	1291	FLOT2 Uncharacterized protein	101	65766	
16	1224	FKBP15 Isoform 1 of FK506-binding protein 15	109	158092	1.539
16	1224	FKBP15 Isoform 2 of FK506-binding protein 15	109	156626	
17	773	RAB10 Ras-related protein Rab-10	202	29447	1.606
18	440	CARS cysteinyl-tRNA synthetase, cytoplasmic isoform c	404	116062	1.551
18	440	CARS cysteinyl-tRNA synthetase, cytoplasmic isoform e	404	111973	
18	440	CARS Isoform 2 of Cysteinyl-tRNA synthetase, cytoplasmic	404	102396	
18	440	CARS Isoform 1 of Cysteinyl-tRNA synthetase, cytoplasmic	404	106485	
18	440	CARS cDNA FLJ38994 fis, clone NT2RI2009259, highly similar to Cysteinyl-tRNA synthetase	404	105124	
19	484	ECH1 Delta(3,5)-Delta(2,4)-dienoyl-CoA isomerase	362	41611	1.547
20	479	FOLR1 Folate receptor alpha	365	34970	1.731
21	871	MRC2 C-type mannose receptor 2	172	184059	1.753
22	1994	RBM7 RNA-binding protein 7	52	35048	1.551
22	1994	RBM7 Uncharacterized protein	52	19833	
22	1994	RBM7 31 kDa protein	52	35135	
22	1994	RBM7 Uncharacterized protein	52	28166	
23	990	MIR1279;CPSF6 Isoform 1 of Cleavage and polyadenylation specificity factor subunit 6	149	64516	2.586
23	990	MIR1279;CPSF6 Uncharacterized protein	149	57667	
23	990	MIR1279;CPSF6 Isoform 2 of Cleavage and polyadenylation specificity factor subunit 6	149	69687	
23	990	MIR1279;CPSF6 Isoform 3 of Cleavage and polyadenylation specificity factor subunit 6	149	57636	
24	1560	MAGEB2 Melanoma-associated antigen B2	78	45465	2.245

**Table 3:** The pathways involved in ING5 overexpression in SGC-7901 cells

Num	Pathway	Diff Proteins	All Proteins	p value
1	Osteoclast differentiation	3 (11.11%)	22 (1.08%)	0.0027207
2	Phagosome	4 (14.81%)	52 (2.56%)	0.0043232
3	Cell adhesion molecules (CAMs)	2 (7.41%)	10 (0.49%)	0.0071602
4	Amyotrophic lateral sclerosis (ALS)	2 (7.41%)	14 (0.69%)	0.0140131
5	ECM-receptor interaction	2 (7.41%)	15 (0.74%)	0.0160374
6	Glycosylphosphatidylinositol(GPI)-anchor biosynthesis	1 (3.7%)	3 (0.15%)	0.0393349
7	Vitamin digestion and absorption	1 (3.7%)	3 (0.15%)	0.0393349
8	Malaria	1 (3.7%)	3 (0.15%)	0.0393349
9	Autoimmune thyroid disease	1 (3.7%)	3 (0.15%)	0.0393349
10	Graft-versus-host disease	1 (3.7%)	3 (0.15%)	0.0393349
11	Allograft rejection	1 (3.7%)	3 (0.15%)	0.0393349
12	Peroxisome	2 (7.41%)	27 (1.33%)	0.0486255

### The relationship between ING5 expression and carcinogenesis or aggressiveness of gastric cancer

**Figure 7:**
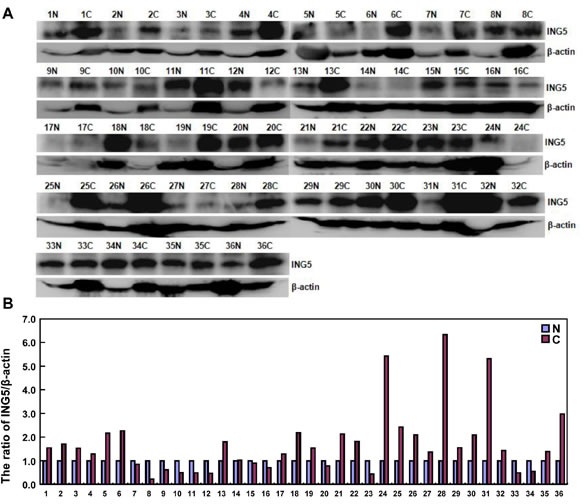

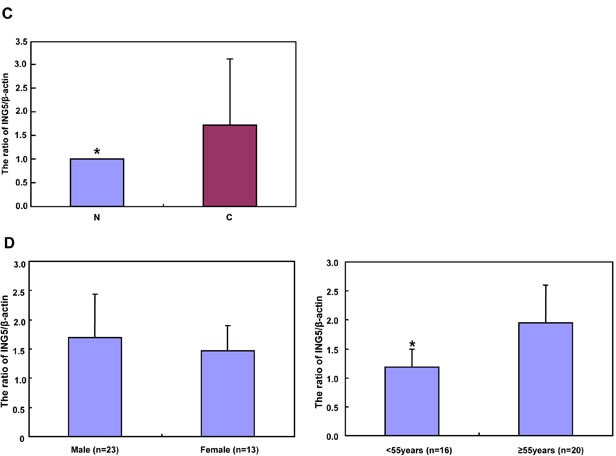

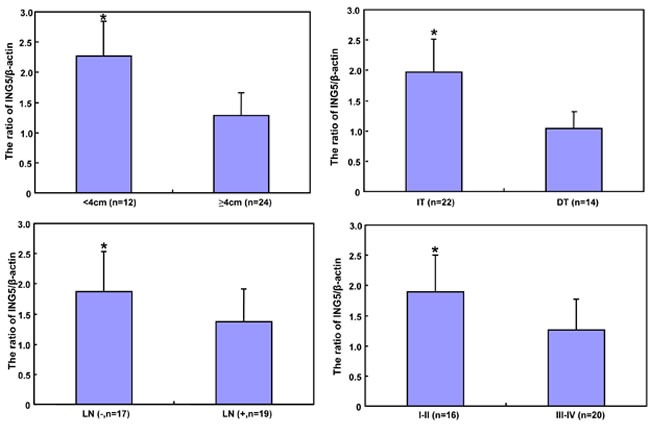
The relationship between ING5 expression and clinicopathological parameters of gastric cancers Tissue lysate was loaded and probed with anti-ING5 antibody (external, 60kDa or internal, 28kDa) with β-actin (42kDa) as an internal control by Western blot. (**A**). The densitometric analysis was performed for ING5 expression in gastric cancer and matched mucosa (**B**). ING5 protein expression level was compared with carcinogenesis and clinicopathological parameters of gastric cancers (**C** and **D**). *p < 0.05. Note: N, matched non-neoplastic mucosa; C, cancer; IT, intestinal-type; DT, diffuse-type; LN, lymph node metastasis.

According to densitometric analysis (Figure 7A and 7B), ING5 expression was increased in 24 cases (66.7%) when compared with matched mucosa, and decreased in 12 cases (33.3%). Statistically, ING5 expression was higher in gastric cancer than the matched mucosa (Figure 7C, *p* < 0.05). As summarized in Figure 7D, ING5 expression was negatively correlated with tumor size, lymph node metastasis, and TNM staging (*p* < 0.05). ING5 was more expressed in elder than younger cancer patients (*p* < 0.05). There was a higher expression of ING5 in intestinal-than diffuse-type carcinomas (*p* < 0.05),

### ING5 suppresses the growth and lung metastasis of gastric cancer cells

**Figure 8:**
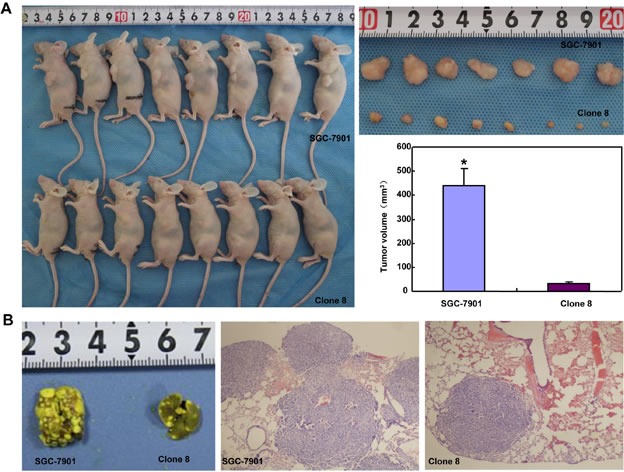

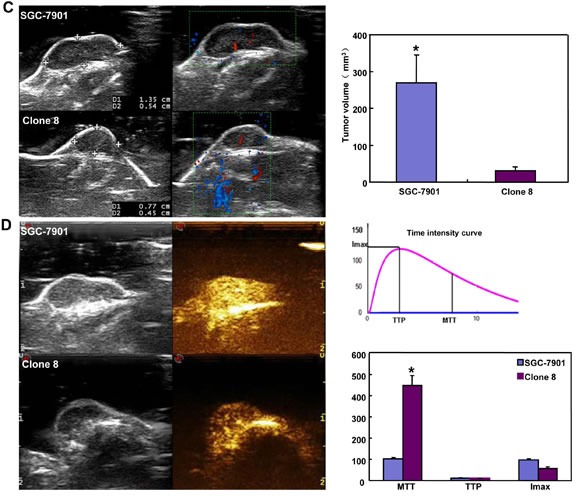

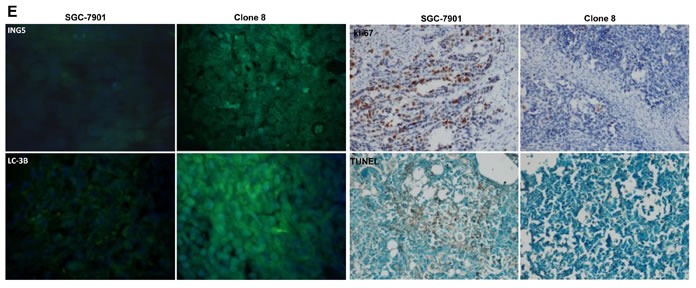
ING5 suppresses the growth and lung metastasis of SGC-7901 cells in nude mice The growth of SGC-7901 cells were faster by measuring tumor volume (**A**) and easier to metastasize into the lung (**B**) than their ING5 transfectants. Ultrasonic examination showed that both tumor volume (**C**) and blood supply (**D**) were larger in SGC-7901 than ING5 transfectants. The transfectants showed a higher expression of ING5 and LC-3B, a lower ki-67 immunoreactivity, and a weaker signal of TUNEL (**E**). *, *p* < 0.05; MTT, mean transit time; TTP, time to peak; Imax, maximum intensity.

The tumor volume of SGC-7901 xenografts was larger, heavier and of more blood supply than those of ING5 transfectants by ruling, ultrasonic imaging and contrast-enhanced ultrasonic imaging respectively (Figure 8A, 8C and 8D, *p* < 0.05). It was the same for tumor number and size of lung metastasis (Figure 8B). SGC-7901 transfectants showed lower proliferation evidenced by ki-67 marker, more authophagy by LC-3B staining, and weaker apoptosis by TUNEL than the control (Figure 8E).

## DISCUSSION

The hypoexpression of nuclear ING5 protein and its cytoplasmic translocation were observed in oral squamous cancer [10], HNSCC [11], colorectal [12] and gastric [13] cancers. However, ING5 overexpression was found in gastric cancer in comparison to matched mucosa by Western blot, which might be attributable to its nucleocytoplasmic translocation from NLS mutation, ING5 expression in stromal cells and a high karyoplasmic ratio of cancer cells. The pEGFP-N1-ING5 transfection and nucleocytoplasmic fraction demonstrated that ING5 protein was localized in the nuclei of gastric cancer or epithelial cells, indicating that the translocating system of ING5 protein works well in the cells. In line with immunohistochemical findings of nuclear ING5 [12], we found that ING5 expression level was negatively linked to tumor size, lymph node metastasis, and clinicopathological staging, indicating that ING5 protein mainly reflected its nuclear product and might be employed to indicate the aggressiveness of gastric cancers [13]. ING5 overexpression was *in vitro* found to decrease migration, invasion and lamellipodia formation with MMP-9 hypoexpression, and *in vivo* suppressed lung metastasis of gastric cancer cells, suggesting that ING5 might inhibit invasion and metastasis of gastric cancer by reducing MMP-9 expression. Moreover, ING5 overexpression was demonstrated to cause the differentiation of SGC-7901 cells evidenced by enhanced ALP activity and remarkable tight junctions, supporting a higher expression of ING5 protein in intestinal-than diffuse-type carcinomas by Western blot and immunohistochemistry [13].

There *in vivo* and *vitro* appeared a low proliferation in SGC-7901 transfecants in comparison to the control. Reportedly, Cyclin E and D1 activate Cdks and induce the transition between G_1_ and S phase [14]. c-jun is required for progression through the G_1_ phase, and c-jun null cells show increased G_1_ arrest [15]. Cyclin B1-Cdk1 is involved in the early events of mitosis, and CDC25B activates the cyclin dependent kinase CDC2 for entry into mitosis [16]. It is believed that 14-3-3 sequesters Cdc25C to the cytoplasm to prevent the interactions with Cyclin B-Cdk1 necessary for G_2_/M transition [17]. In SGC-7901 transfectants, ectopic ING5 expression caused G_1_ phase arrest, which was positively linked to 14-3-3 overexpression, Cdk4 and c-jun hypoexpression. The stronger expression of cdc-2, Cyclin B1 and cdc25B might be compensatory for the low proportion of G_2_-phase cells.

Interestingly, ING5 overexpression could suppress the apoptosis of SGC-7901 by increasing mitochondrial membrane potential. In apoptosis, Bcl-2 can interact with Bax on the mitochondrial membrane to suppress the apoptosis because Bax is believed to open the mitochondrial voltage-dependent anion channel for apoptosis [18]. Phosphorylated BAD can be complexed by 14-3-3 protein, preventing the association of BAD with Bcl-xL and Bcl-2, and inhibiting apoptosis [19]. XIAP might function as apoptotic inhibitor by binding to and suppressing caspase activation [20]. Our investigation indicated that lower expression Bax, higher expression of Bcl-2, survivin and 14-3-3 induced apoptotic inhibition of ING5 via mitochondrial pathway. Reportedly, Akt1 inactivates Caspase 9 by phosphorylation on ser196. After phosphorylation by Akt1, BAD is released from Bcl-2 or Bcl-xL and loses its pro-apoptotic effect [21]. The high level of phosphoralyted Akt in ING5 transfectants might be responsible for the apoptotic inhibition.

Wnt signaling pathway can inhibit GSK-3-mediated phosphorylation of β-catenin allowing β-catenin to the nucleus, where the interaction of β-catenin with TCF family transcription factors regulates gene expression [22]. Here, ectopic ING5 expression was found to enhance the mRNA and protein expression of β-catenin in SGC-7901, as well as its phosphorylation. It was true for β-catenin-targeting TCF-4 promoter and TCF-mediated gene transcription, such as, *c-myc*, *VEGF, survivin* and *Cyclin D1* [23]. Therefore, it is hypothesized that ING5 overexpression activates the β-catenin pathway, finally to up-regulate the mRNA expression of target genes via TCF-4 transcriptional factor.

Although autophagy is a process of sequestering cellular cytosol and intracellular organelles into autophagosome, and phagocystosis is engaged in sequestering cellular exterior, both events are capture and digestion of material and display similar morphological appearances [24]. The top canonical pathways included phagosome and cell adhesion molecules in ING5 transfectants. Reportedly, cell adhesion molecules, like E-cadherin, are delivered via secretory vesicles as cargo to sites of polarity and involved in exocytosis [25], consistent with our results. During autophagy, mTOR kinase signaling pathway positively regulates the formation of ATG13-ULK1-RB1CC1 complex. Subsequently, autophagosome formation is induced by III PI3K, Beclin-1 and ATG-14, finally to promote ATG12-ATG7 conjugation [26]. Here, it was found that ING5 overexpression *in vivo* and *vitro* induced the autophagy of SGC-7901 cells with Beclin 1 overexpression, indicating that ING5-induced autophagy was dependent on beclin-1 and belonged to canonical pathway.

The chemoresistance of ING5 transfectants to triciribine, paclitaxel, cisplatin, SAHA, MG132 and parthenolide was positively correlated with their apoptotic induction. Particularly, the up-regulated expression of drug resistance genes might contribute to ING-5-mediated chemoresistance, including *MDR1*, *GRP78*, *GRP94*, *IRE*, *CD147*, *FBXW7*, *TOP1*, *TOP2*, *MLH1*, *MRP1*, *BRCP1*, and *GST-π* [27-31]. Akt/PI3K/p70S6K pathway is reported to promote cellular survival and inhibits apoptotic processes [32]. In addition, novel PKC δ and ζ are activated through DAG, and suppress apoptosis [33]. The overexpression of PI3K, p-Akt, p70S6K and nPKC in ING-5-overexpressing SGC-7901 demonstrated that Akt/PI3K/p70S6K activation and nPKC overexpression might enhance the apoptotic and chemotherapeutic resistance of gastric cancer cells.

NF-κB is a protein complex that controls transcription (e.g. cytokines) and protects cancer cells from apoptosis [34]. Here, ING5 overexpression was found to activate NF-κB pathway by upregulating its transcriptional activity and expression, indicating that NF-κB might have impact on ING5-mediated drug resistance. It was documented that specificity protein 1(Sp1) and Stat5a/b belong to transcription factor and mediate the signals of cytokines [35, 36]. In SGC-7901 transfectants, overexpression of NF-κB, Sp-1 and stat5a/b might be responsible for the higher expression of ILs. In proteomic analysis, up-regulated genes belong to MAPK, NF-κB, histone and cholesterol metabolism (APO1), among which the former two are positively linked to the osteoclast differentiation. In addition, the down-regulated genes are involved in the cell division cycle and apoptosis (e.g. CCAR), chemical modification of RNA (e.g. CARS and SNORD19B) and mobility (e.g. PACSIN3, MAP1B and FLOT2). Therefore, ING5 overexpression induced cell cycle arrest, apoptotic resistance, and suppressed invasion and migration as observed in our data.

In conclusion, ING5 overexpression might suppress the proliferation, migration and invasion, induce autophagy and differentiation, and mediate apoptotic and chemotherapeutic resistance of gastric cancer cells. ING5 overexpression might activate β-catenin, NF-κB and Akt pathways in gastric cancer. Therefore, it has to be careful to employ ING5 as a target of gene therapy for gastric cancer.

## MATERIALS AND METHODS

### Cell culture

Gastric cancer cell lines, MKN28, AGS, BGC-823, MGC-803, MNK45 and SGC-7901, KATO-III, HGC-27, GT-3 TKB and STKM2, SCH and gastric epithelial cell line GES-1 come from Japanese Physical and Chemical Institute, Tokyo, Japan, Beijing Institute for Cancer Research, Beijing, China, and Cell bank of Chinese Academy of Sciences, Shanghai, China, respectively. They were maintained in RPMI 1640 or Ham F12 medium supplemented with 10% fetal bovine serum (FBS), 100 units/mL penicillin, and 100μg/mL streptomycin, in a humidified atmosphere of 5% CO_2_ at 37°C. To check the drug sensitivity, we exposed cells to triciribine (Akt inhibitor), cisplatin (a platinum-containing DNA crosslinker), paclitaxel (a mitotic inhibitor), MG132 (Enzo, proteosome inhibitor), SAHA (a HDAC inhibitor) and parthenolide (a sesquiterpene lactone). Cells were fractionated into cytosolic and nuclear fraction using NE-PER Nuclear and Cytoplasmic Extraction Reagents (Pierce).

### Plasmid construction and transfection

Plasmid pCDNA3.1-ING5 (kindly presented by Prof. Côté) was used to construct of the COOH-terminally GFP-tagged ING5 plasmid (pEGFP-N1-ING5). The amplified ING5 DNA was digested and inserted into pEGFP-N1 (Clontech, USA) between *BamH* I and *Hind* III. SGC-7901 cells were transfected with pEGFP-N1-ING5, pEGFP-N1 vector or pEGFP-N1-LC-3B at 24 h after seeding on dishes, or selected by G418. siRNAs were also transfected to silence ING5 expression in GT-3TKB. The target sequences of ING5 were 3′-GAAAAGAGGAAGAAGAAGT-5′ (sense) and 3′-ACTTCTT CTTCCTCTTTTC-5′ (anti-sense).

### Proliferation assay

Cell Counting Kit-8 was employed to determine the number of viable cells. In brief, 2.5 × 10^3^ cells/well were seeded on 96-well plate and allowed to adhere. At different time points, 10 μL of CCK-8 solution was added into each well of the plate and the plates were incubated for 3 h in the incubator, and then measured at 450 nm.

### Cell cycle analysis

1 × 10^6^ cells were collected, washed by PBS twice and fixed in cold 10mL 75% ethanol for more than 2 h. And then, cells were washed by PBS and incubated with RNase at 37°C for 1 h. The tube with cells was added by PI to 50 μg/mL and incubated at 4°C in the dark for 30 min. Finally, FACS was employed to examine the PI signal.

### IdU/CldU-staining procedure for detection of DNA replication

DNA replication foci were visualized by incorporation of chlorodeoxyuridine (CldU) and iododeoxyuridine (IdU). Briefly, cells were labeled for 1 h with 10 μM CIdU and then 10 μM IdU (Chemos GmbH). Primary anti-CldU (Abcam) and then Alexafluor 488-conjugated anti-rat (Invitrogen) antibodies were added to the slides, and incubated for 1 h respectively. Then, primary mouse anti-IdU (Sigma) and then Alexafluor 594-conjugated anti-mouse (Invitrogen) antibodies were added to the slides, and incubated for 1 h respectively. Images were visualized with a laser confocal microscope.

### Apoptosis assay by flow cytometry

Flow cytometry was performed with 7-amino-actinomycin (7-AAD) and phycoerythrin (PE)-labeled Annexin V (BD Pharmingen, USA) to detect phosphatidylserine externalization as an endpoint indicator of apoptosis as the protocol recommends.

### Wound healing assay

Cells were seeded at 1.0×10^6^ cells/well in 6-well culture plates. After they had grown to confluence, the cell monolayer was scraped with a pipette tip to create a scratch, washed by PBS for three times and cultured in the FBS-free medium. Cells were photographed and the scratch area was measured using Image software.

### Cell migration and invasion assays

For migration assay, 2.5 × 10^5^ cells were resuspended in serum-free RPMI 1640, and seeded in the control-membrane insert on the top portion of the chamber (BD Bioscience). The lower compartment of the chamber contained 10% FBS as a chemo-attractant. After incubated for 24 h, cells on the membrane were scrubbed, washed with PBS and fixed in 100% methanol and stained with Giemsa dye. For invasive assay, the procedures were the same as above excluding the matrigel-coated insert (BD Bioscience).

### Alkaline phosphatase (ALP) activity

ALP activity was used as a marker of colorectal differentiation. The cells were harvested, broken and subjected to the determination of ALP activity using Diagnostics ALP reagent (Sigma, USA). The protein content of the samples was determined Biorad protein assay kit (Biorad, USA). ALP activity was calculated as U per g of protein.

### Transmission electron microscopy

Specimens were immersed in 2% cacodylate-buffered glutaraldehyde for 6 h. They were then rinsed in cacodylate buffer supplemented with 15% sucrose, post-fixed with 1% phosphate-buffered OsO4 (pH 7.4) for 2 h, dehydrated with alcohol, clarified in propylene oxide, and embedded in Epon. Ultrathin sections were made with ultramicrotome, stained with uranyl acetate, followed by a saturated solution of bismuth subnitrate and finally examined under a JEOL 1010 electron microscope.

### Immunofluorescence

Cells were grown on glass coverslips, washed twice with PBS, fixed with PBS containing 4% formaldehyde for 10 min, and permeabilized with 0.2% Triton X-100 in PBS for 10 min. After washing with PBS, cells were incubated overnight at 4 °C with the antibody against ING5 (Proteintech) or LC-3B (Cell Signal). They were then washed with PBS and incubated with Alexa Fluor 488 IgG (Invitrogen). Alexa Fluor® 568 phalloidin (invitrogen) was employed to observe the lamellipodia. Nuclei were stained with DAPI (Sigma). Finally, coverslips were mounted with SlowFade® Gold reagent (invitrogen) and observed under laser confocal scanning microscope.

### Luciferase reporter assay

To evaluate the β-catenin/TCF-4 and NF-κB transcriptional activity, we transiently co-transfected TCF-4 promoter reporter gene plasmid, pGL-[1306]TCF4-Luc, or TCF-mediated transcription reporter gene plasmid, pGL3-OT and pGL3-OF, using Lipofectamine 2000. Luciferase activity was measured 48 h after transfection using dual-luciferase reporter assay system. The Renilla luciferase activity was used as an internal control. TCF-mediated gene transcription activity was determined by the ratio of pGL3-OT to pGL3-OF luciferase activity, which was normalized to Renilla luciferase activity of pRL-TK. TCF-4 and NF-κB promoter activity was determined by the value of pGL-[1306]. TCF4-Luc and NF-κB luciferase activity, which was also normalized by Renilla luciferase activity of pRL-TK.

### Proteomic analysis by iTRAQ labeling and 2D Nano-LC-MS/MS

For iTRAQ analysis, the protein samples were extracted from SGC-7901 and its ING5 transfectants (Clone 8) in RIPA lysis buffer containing cocktail protease inhibitors (Roche). Two hundred micrograms of each sample were digested and labeled with the following iTRAQ labels. Samples were separated by strong cation exchange and sequentially analyzed by two-dimensional LC-MS/MS. Data were acquired under the information-dependent acquisition mode, with dynamic exclusion set to exclude any m/z values that had been picked for MS/MS scan. The bioinformatics analysis of the differentially expressed proteins was performed with Ingenuity Pathways Analysis (IPA) software (version 6.3, Ingenuity Systems, Redwood City, CA; http://www.ingenuity.com). Details are provided in the Supporting Information.

### Subjects and pathology

Gastric cancers and matched mucosa were collected from the First Affiliated Hospital of Liaoning Medical University (China) between 2012 and 2013 and frozen in −80°C until protein and RNA extraction. The patients with gastric cancer were 23 men and 13 women (34-81years, mean=56.9years). None of the patients underwent chemotherapy, radiotherapy or adjuvant before surgery. They all provided written consent for use of tumor tissue for clinical research and our University Ethical Committee approved the research protocol. TNM staging for gastric cancer was evaluated according to Union Internationale Contre le Cancer system. Histological architecture of gastric cancer was expressed in terms of Lauren's classification.

### Xenograft models

Locally bred BALB/c nude mice were maintained under SPF condition, and food and water were supplied ad libitum. All procedures involving animals were performed in compliance with the Committee for Animal Experiments guidelines on animal welfare of Liaoning Medical University. Subcutaneous xenografts were established by injection of 1× 10 ^6^ cancer cells per mouse at the age of 6–8 week (n=10/group). Tail vein assay of cancer metastasis was performed by intravenous injection of 1× 10^6^ cancer cells (n=5/group). Tumor volume was measured using calipers, and calculated as follows: length × width × depth × 0.52. After 2 to 3 weeks of post-injection, we euthanized mice by CO_2_ asphyxiation and removed tumors and lungs, rinsed in tap water, and fixed in 4% formaldehyde.

### Contrast-enhanced ultrasonic imaging

Ultrasound images of xenograft tumor were obtained on anaesthetized nude mice using a Philips iU22 (Bothell, WA, USA) ultrasound scanner with the curve-linear array probe C5-2. The imaging parameters were: power modulation (PM 3 pulses) transmit frequency 1.7MHz at low transmit power (mechanical index,0.06), approximately 7–10 frames per s and one focus well below the level of the target lesion to ensure a more uniform pressure field. At each imaging session, tumor volumes were assessed in fundamental B-mode imaging using calipers. They were calculated using the following formula: length × width × depth × 0.52. During each imaging session, mice were subjected to an intravenous bolus of 0.2 mL of contrast agent (sulfur hexafluoride, SonoVue; Bracco, Italy) in 2 s. Subsequently, color Doppler flow imaging was employed to visualize contrast refilling in tumor. All the experiments were recorded on digital videotapes (Digital Video Recorder Sony, GV-D900E PAL). Time intensity and flash replenishment curves were generated by quantitative analysis performed in a region of interest using the software Pulse.

### Reverse transcriptase-polymerase chain reaction (RT-PCR)

Total RNA was extracted from gastric cancer and epithelial cell lines using QIAGEN RNeasy mini kit (Germany). Total RNA were subjected to cDNA synthesis using AMV reverse transcriptase and random primer (Takara). General and real-time RT-PCR amplification was performed using Hotstart Taq polymerase (Takara) and SYBR Premix Ex Taq ^TM^ II kit (Takara) respectively. According to the Genbank, oligonucleotide primers were designed and shown in supplementary Table 1.

### Western blot

Protein assay were performed using Biorad protein assay kit. The denatured protein was separated on SDS-polyacrylamide gel and transferred to Hybond membrane, which was then blocked overnight in 5% milk in TBST. For immunoblotting, the membrane was incubated with the primary antibody (Supplement Table 2). Then, it was rinsed by TBST and incubated with IgG-conjugated to horseradish peroxidase (DAKO). Bands were visualized by ECL-Plus detection reagents (Santa cruz). The densitometric quantification was performed with a β-actin control using Scion Image software.

### Terminal digoxigenin-labeled dUTP nick-end labeling (TUNEL)

Cell apoptosis was assessed using TUENL, a method that is based on the specific binding O-TdT to the 3-OH ends of DNA, ensuring the synthesis of a polydeoxynucleotide polymer. For this purpose, ApopTag Plus Peroxidase In Situ Apoptosis Detection Kit (Chemicon) was employed according to the recommendation. Omission of the working strength TdT enzyme was considered as negative control.

### Statistical analysis

The results are representative of 3 different experiments, and data are expressed as mean ± standard deviation. Mann-Whitney U was employed to differentiate the means. *P* < 0.05 was considered as statistically significant. SPSS 10.0 software was employed to analyze all data.

## SUPPLEMENTARY TABLES


