# Supplementary material for: ING5 suppresses proliferation, apoptosis, migration and invasion, and induces autophagy and differentiation of gastric cancer cells: a good marker for carcinogenesis and subsequent progression
Source: Oncotarget. 2015 Mar 30;6(23):19552–79. doi: 10.18632/oncotarget.3735 (PMC4637305; doi:10.18632/oncotarget.3735)
Supplement: Supplementary file 1 [file oncotarget-06-19552-s001.pdf]

ING5 suppresses proliferation, apoptosis, migration and invasion, and induces autophagy and differentiation of gastric cancer cells: a good marker for carcinogenesis and subsequent progression

Supplementary Material

Supplementary Table 1 The primers used in RT-PCR

	Names	Primer' s sequence	Distribution	AT(°C)	Product size(bp)	Extension time(s)
1	*ING5*	F: 5'- GGGAGATGATTGGCTGTG-3'<br>R: 5'-CCTTTGGGTTTCGTGGTA-3'	NM_032329.4<br>614-759	60	146	34
2	*CyclinE1*	F: 5'-GGATGTTGACTGCCTTGA-3'<br>R: 5'-CGCACCACTGATACCCT-3'	BA000005<br>1044-1150	60	107	34
3	*cdc2*	F: 5' –GGGCACTCCCAATAA-3'<br>R: 5'-GATGCTAGGCTTCCTG-3'	XM_572099<br>631-723	60	93	34
4	*Bcl-2*	F: 5'-GCCTTCTTTGAGTTCGGTGGG-3'<br>R: 5'-TGTGCAGGTGCCGGTTCAG-3'	DQ926871<br>938-1052	60	115	34
5	*Bax*	F: 5'-GATTGCCGCCGTGGAC-3'<br>R: 5'-GCCCCAGTTGAAGTTGC-3'	DQ926869<br>306-393	60	88	34
6	*14-3-3*	F: 5'-CAAAGACAGCACCCTCA-3'<br>R: 5'-TTCTGCCGCATCACAT-3'	XM_010379682.1<br>845-935	60	91	34
7	*Raptor*	F: 5' –GGAGCGCCCGCTATTT-3'<br>R: 5'-GTCGTCCAATCTCGTAATGC-3'	BC136654<br>3690-3850	60	161	34
8	*mToR*	F: 5'-CGCTGTCATCCCTTTATC-3'<br>R: 5'-TTCTTCTCCCTGTAGTCCC-3'	NM_004958<br>2092-2187	60	96	34
9	*Akt2*	F: 5'-CGGCTCCTTCATTGGG-3'<br>R: 5'-GCTGGCATTCTGCTACGG-3'	HUMAKT2A<br>404-492	60	89	34
10	*Akt3*	F: 5'-GACATTCTGTGGCACTC-3'<br>R: 5'-TCTCATGGTCCTGGTT-3'	NM_005465<br>1065-1210	60	146	34
11	*IL-1*	F: 5'- GACGCCCTCAATCAAAGT-3'<br>R:5'-CTTGGGCAGTCACATACA-3'	KJ891448.1<br>477-654	60	178	34
12	*IL-2*	F: 5'- GACTTTACTGCTGGATTT-3'<br>R: 5'- ATTGCTGATTAAGTCCCT-3'	NM_000586.3<br>160-379	60	220	34
13	*IL-4*	F: 5'- CAGTTCTACAGCCACCAT-3'<br>R: 5'- CTGGTTGGCTTCCTTCAC-3'	NM_172348.2<br>249-407	60	159	34
14	*IL-10*	F: 5'- TCAGGGTGGCGACTCTAT-3'<br>R:5'-TGGGCTTCTTTCTAAATCGTT-3'	NM_000572.2<br>601-799	60	199	34
15	*IL-17*	F: 5'- GAAGGCAGGAATCACAAT-3'<br>R:5'-ATCGGTTGTAGTAATCTG-3'	NM_002190.2<br>108-253	60	146	34
16	*CyclinD1*	F: 5'-TGCCACAGATGTGAAGTTCATT-3'<br>R: 5'-CAGTCCGGGTCACACTTGAT-3'	NG_000002<br>776-937	60	162	34
17	*Survivin*	F: 5'-TTCTCAAGGACCACCGCATC-3'<br>R: 5'-AGCCTTCCAGCTCCTTGAAG-3'	DQ508252<br>159-320	60	162	34
18	*C-myc*	F: 5'-AGCGACTCTGAGGAGGAACA-3'<br>R: 5'-TCCAGCAGAAGGTGATCCA-3'	X00676<br>1318-1425	60	108	34
19	*VEGF*	F: 5'-GCGCTCGGTGCTGGAATTTG-3'<br>R: 5'-TAGAGCAATCTCCCCAAGCCGTCG-3'	NM_001204384.1<br>118-276	60	159	34
20	*IRE1*	F: 5'-ACTGGCTTCTGATAGGAC-3'<br>R: 5'-GATGTTTGGGTAGATTGTT-3'	XM_511585.5<br>2251-2337	60	87	34
21	*GRP94*	F: 5'-TGACGATGAAGTTGATGTGGAT-3'<br>R: 5'-CATCATTCTGTTAACTTCGGCTT-3'	XM_003832567.2<br>245-440	60	196	34
22	*MDR-1*	F: 5'-ACACCTGGGCATCGT-3'<br>R:5'-TATTAGGCAGTGACTCGA-3'	NM_000927<br>3826-3983	60	158	34
23	*GRP78*	F: 5'-GTTCTTGCCGTTCAAGGTGG-3'<br>R:5'-TGGTACAGTAACAACTGCATG-3'	FJ436356<br>600-780	60	181	34
24	*CD147*	F: 5'-TACTCCTGCGTCTTCCTCC-3'<br>R:5'-TGCGAGGAACTCACGAAG-3'	KJ896510.1<br>318-556	60	239	34
25	*FBXW7*	F: 5'-AGATGGACCAGGAGAGTG-3'<br>R:5'-CTTGCATGGTTTCTTTCC-3'	XM_009448414.1<br>554-771	60	218	34
26	*TOP1*	F: 5'-AAAGATCGAGAACACCGG-3'<br>R:5'-TGTTTGGTCTTCTCCTTCT-3'	XM_004062154.1<br>335-456	60	122	34

27	*TOP2*	F: 5'-AAAATGAAGATGCTAAGAAAAGACT-3'<br>R:5'-GTACAAACCAGGAACAAAAGTGACT-3'	XM_003315476.2<br>226-413	60	188	34
28	*MLH1*	F: 5'-TGCAAAATCCACAAGTATTCA-3'<br>R:5'-TGTATGCACACTTTCCATCAG-3'	XM_009445169.1<br>211-467	60	257	34
29	*MRP1*	F: 5'-TTTCAGAACACGGTCCTCG-3'<br>R:5'-TGGGCTGACCAGAAACACT-3'	XM_009430367.1<br>118-351	60	234	34
30	*BCRP*	F: 5'-GACAGCTTCCAATGACCTGAA-3'<br>R: 5'-CAGGATGGCGTTGAGACC-3'	XM_005263356<br>285-456	60	172	34
31	*GST π*	F: 5'- CGGGCAAGGATGACTATGTGA -3'<br>R: 5'- GGGCTAGGACCTCATGGATCA -3'	XM_001152516<br>585-746	60	162	34
32	*GAPDH*	F: 5'-CAATGACCCCTTCATTGACC-3'<br>R: 5'- TGGAAGATGGTGATGGGATT-3'	NM_ 002046.3<br>201-335	60	135	34

Supplementary Table 2 The primary antibodies used in Western blot.

	Antibody	Species	Dilution	Company	Code number
1	ING5	rabbit	1:1000	proteintech	10665-1-AP
2	p21 (F-5)	mouse	1:500	santa cruz	sc-6246
3	Cyclin E1(C-19)	rabbit	1:500	santa cruz	sc-198
4	Cyclin D1(H-295)	rabbit	1:500	santa cruz	sc-753
5	Cdk4 (C-22)	rabbit	1:500	santa cruz	sc-260
6	Cyclin B1 (GNS1)	mouse	1:500	santa cruz	sc-245
7	Cdc2 p34 (B-6)	mouse	1:500	santa cruz	sc-8395
8	p-Cdc2 p34 (Tyr 15)	rabbit	1:500	santa cruz	sc-7989
9	c-Jun (H-79)	rabbit	1:500	santa cruz	sc-1694
10	Cdc25B (H-85)	rabbit	1:500	santa cruz	sc-5619
11	Cdc25C (H-6)	mouse	1:500	santa cruz	sc-13138
12	Bcl-2 (C 21)	rabbit	1:500	santa cruz	sc-783
13	Bax (B-9)	mouse	1:500	santa cruz	sc-7480
14	AIF (E-1)	mouse	1:700	santa cruz	sc-13116
15	XIAP (H-202)	rabbit	1:500	santa cruz	sc-11426
16	14-3-3（H-8）	mouse	1:500	santa cruz	sc-1657
17	c-myc (9E10)	mouse	1:500	santa cruz	sc-40
18	Beclin 1	rabbit	1:2000	abcam	Ab51031
19	ATG7(N-20)	goat	1:500	santa cruz	sc-8668
20	ATG14	rabbit	1:1000	cell signaling	#5504
21	Akt1/2/3 (H-136)	rabbit	1:500	santa cruz	sc-8312
22	p-Akt1/2/3 (Ser473)	rabbit	1:500	santa cruz	sc-101629
23	PI3-Kinase p100 (D-4)	mouse	1:500	santa cruz	sc-8010
24	p70 S6 kinase β (N-19)	goat	1:500	santa cruz	sc-9379
25	PKC ζ (C-20)	rabbit	1:500	santa cruz	sc-216
26	PKC δ (C-17)	rabbit	1:500	santa cruz	sc-213
27	MMP-9 (H-129)	rabbit	1:500	santa cruz	sc-10737
28	p38α/β (H-147)	rabbit	1:500	santa cruz	sc-7149
29	Sp1(PEP2)	rabbit	1:500	santa cruz	sc-59
30	SIRT1(H-300)	rabbit	1:500	santa cruz	sc-15404
31	p-Stat5a/b (Tyr 694)	rabbit	1:500	santa cruz	sc-101806
32	Ac-Histone H3 (Lys 9/14)	rabbit	1:1000	santa cruz	sc-8655-R
33	Ac-Histone H4 (Lys 8)	rabbit	1:1000	santa cruz	sc-8660-R
34	β-catenin（C-18）	goat	1:500	santa cruz	sc-1496
35	NF-κB p65(A)	rabbit	1:500	santa cruz	sc-109
36	β-actin(C4)	mouse	1:2000	santa cruz	sc-47778
